# Sports-related injuries sustained by officers of the State Fire Service on duty – nationwide 7-year follow-up

**DOI:** 10.3389/fpubh.2023.1204841

**Published:** 2023-07-07

**Authors:** Łukasz Dudziński, Mariusz Panczyk, Tomasz Kubiak, Tomasz Milczarczyk

**Affiliations:** ^1^John Paul II Academy in Biała Podlaska, Biała Podlaska, Poland; ^2^Medical University of Warsaw, Warsaw, Poland; ^3^Academy of Applied Science Mieszka I in Poznan, Poznań, Poland; ^4^The main headquarters of the State Fire Service, Warsaw, Poland

**Keywords:** State Fire Service, sports activities, physical activity, injuries, contusions to injuries

## Abstract

**Aim:**

The accident rate in the State Fire Service from 2015 to 2021 related to sports activities was analyzed in relation to the regions of the country per year.

**Materials and methods:**

The study included analysis of data from the SFS Headquarters – Department for Occupational Health and Safety and Preventive Health. Data collected from across the country in the form of an annual analysis of the accident. The reports included such information as: the number of accidents, the cause and circumstances of accident (injury), with a breakdown listing individual and group accidents.

**Results:**

During the observation period, about 30,000 officers were on duty in the SFS, of which about 20% were on daily (8-h) duty, and 80% were on shift (24-h) duty. Between 2015 and 2021, there were *N* = 11,332 (Mean: 1617.4; SD: 284.1) accidents in SFS. Total accident covers individual and mass accidents. The number of sports injuries was *N* = 4,254 (Mean: 532.2; SD: 137.9).

**Conclusion:**

There is a need for comprehensive approach to physical training in the firefighter population. Physical activity should be continuous and systematic strengthening of the whole body. Sports activities should begin with performing thorough warm-ups. It is necessary to maintain facilities, premises, equipment and technical devices in a condition that sets the ground for doing sports safely and in a hygienic manner. Most of the sports injuries sustained by firefighters are related to team sports.

## Introduction

Firefighters are a high-risk occupational group with many hazards that may adversely affect their health. Threats to firefighters are mainly related to rescue and firefighting operations, as well as exercises, training, and sports activities related to the service (1, 2).

The main task of a firefighter is to protect life, health and property, and to eliminate sources of danger: fire, natural disaster, local hazards (road accidents, construction disasters). Interventions of firefighters require specialist knowledge and skills: diving rescue, high altitude rescue, elimination of chemical and radiation hazards. Actions carried out by firefighters often involve exhausting rescue operations lasting many hours (3, 4).

The physical fitness of firefighters is important in the proper and effective conduct of rescue and firefighting operations. Officers of the State Fire Service (SFS) carry out operations under time pressure in the danger zone (fire, explosive zone, construction disasters). Their agility and fitness often determines how fast they are able to reach person in need of help, and also how rapidly can proceed evacuation from the danger zone, which very often translates into the effectiveness of rescue operations and saving endangered life and health (5).

Every firefighter is obliged to preserve physical fitness at an appropriate level that will let him or her 24 h a day, and in all, often adverse weather conditions, carry out rescue operations at the highest level. Among the unfavorable conditions in the working environment of firefighters are extreme temperatures, humidity, limited visibility, the need to carry objects of considerable weight (such as rescue equipment), and, as part of evacuation, the carrying of persons with limited ability to move independently (6).

An important part of a firefighter’s course of duty embedded in in-service training is physical education. Not just firefighters, but already firefighter candidates should present appropriate level of physical fitness. Admission to the SFS is possible in two ways; through candidate service in a fire school (Warsaw, Krakow, Poznan, Czestochowa), or preparatory service as part of the recruitment of candidates organized by SFS organizational unit. Physical fitness tests are mandatory regardless of the chosen way of admission to the fire service (7).

Firefighters serving in the SFS have daily contact with sports. Sports competitions in the SFS are held annually (cyclically) in accordance with a pre-planned annual schedule (calendar of sports events) approved by the Commander-in-Chief (CC) of the SFS. In line with the calendar, firefighters are offered a wide range of options for choosing which discipline to participate in. Sports competitions for firefighters can be local (municipal or district outreach), provincial or national. The schedule of competitions is very extensive, providing for a number of sports (individual, team), seasonal disciplines (summer, winter), local memorials organized in memory of persons of merit in firefighting, or associated with the commemoration of an important date. Among the competitions that have the status of national championships for firefighters are cross-country skiing, alpine skiing, triathlon, table tennis, volleyball, marathon, beach volleyball, and road cycling. In addition, championships are organized in disciplines typically associated with the firefighting profession reflecting the nature of the physical strain that a firefighter may encounter during rescue and firefighting operations, such as stair running, the Polish Championships in Fire Sports, the hook ladder competition, the Polish Championships of Firefighters – Divers (8, 9).

Injuries related to sports activities are part of the risk, no matter if a given sport activity is professional or amateur (regular or occasional). During the course of their service in the SFS, firefighters are exposed to a number of different hazards, not only during their rescue operations, but also during exercises, training and other activities arising from the scope of their tasks. These hazards have the potential to lead to numerous accidents while on duty. Accordingly, accident prevention is one of the main objectives of preventive health and safety (HSE) activities in the SFS (10, 11).

Marszałek et al. notes that in Poland are no legal regulations on how firefighters should conduct the training they should carry out in order to maintain high physical fitness. The principle of annual checking of the efficiency of firefighters by commanders is in force, but it is up to firefighters whether they take care of their physical condition and with what methods (12).

Popular sports disciplines among Polish firefighters are team sports, football, volleyball, or disciplines related to their specialization [swimming for fire-divers, climbing for firefighters from high-altitude groups (13, 14)].

Sports-related injuries among firefighters are typical of highly traumatic team sports and are mainly related to contact with another competitor. They can occur as acute sudden mechanical injuries, in which the moment of injury and the cause can be determined, and the first symptoms of the injury are pain, swelling or bleeding (15, 16).

### Aim

The accident rate in the State Fire Service from 2015 to 2021 related to sports activities was analyzed in relation to the regions of the country per year.

## Materials and methods

### Design

It was observational retrospective descriptive study. The study included analysis of data from the SFS Headquarters -Human Resources Office. Data collected from across the country in the form of an annual analysis of the accident rate. The reports included such information as: the number of accidents, the injured firefighters, the age and length of service of the injured officers, the cause and circumstances of accident (injury), with a breakdown listing individual and group accidents, the length and type of medical treatment given. Events related to officers’ sports activities were selected from the databases provided.

### Ethical considerations

All personal data of officers and of the SFS organizational units they represent are anonymized and the analysis of health and safety reports complies with the principles of the Declaration of Helsinki, so the opinion of the Bioethics Committee was not requested.

### Data collection

In this study, were analyzed several variables including the number and type of sports accidents by discipline, the year of occurrence of the accidents, and their geolocation data by district. Only injuries to firefighters sustained during service-related sports activities, i.e., while performing their scheduled duty or during sports competitions in which firefighter participated on official assignment (representing his or her organizational unit), were taken into account. Injuries caused by sports activities resulting in a firefighter’s temporary inability to serve resulting from off-duty physical activity were not taken into account. Sports-related injuries to volunteer firefighters (Voluntary Fire Brigade), who also participate in many sports competitions while representing their individual fire protection units (FPUs), were not considered for the analysis.

### Characteristics of the research area

The observations focused on the entire Polish SFS population. In the time period covered by the analysis, the number of SFS officers was about 30,000 (Table 1) (17). The structure of the Polish State Fire Service is made up of 16 provincial fire departments (each of them with district units), firefighting schools, the Scientific and Research Center for Fire Protection (SRCFP) in Józefów, and the Central Museum of Fire Fighting in Mysłowice (18).

**Table 1:** Employment status of officers in the SFS in the years covered by the analysis (as at the end of each year of the analysis).

Analysis year	Employment in SFS
2015	29997
2016	29851
2017	29792
2018	30240
2019	30351
2020	29969
2021	30249
Mean 2015–2021	300641
SD 2015–2021	2161

### Statistical analysis

Descriptive statistics were used to characterize the variables. For quantitative variables, the following measures were calculated: mean (M) and standard deviation (SD). For categorical variables, the following structure measures were calculated: number (n) and frequency (%). The method of spatial data visualization in the form of heat maps was also used, using the intensity of colors depending on the characteristics of the event. The maps show the administrative division of the country into 16 districts. The average number of sports accidents in individual disciplines per 1,000 firefighters in subsequent years was compared using one-way ANOVA. The null hypotheses were verified at the assumed type I error rate of 0.05. All statistical calculations were performed using STATISTICA software version 13.3 (CA, United States).

### Results

**Figure 1:**
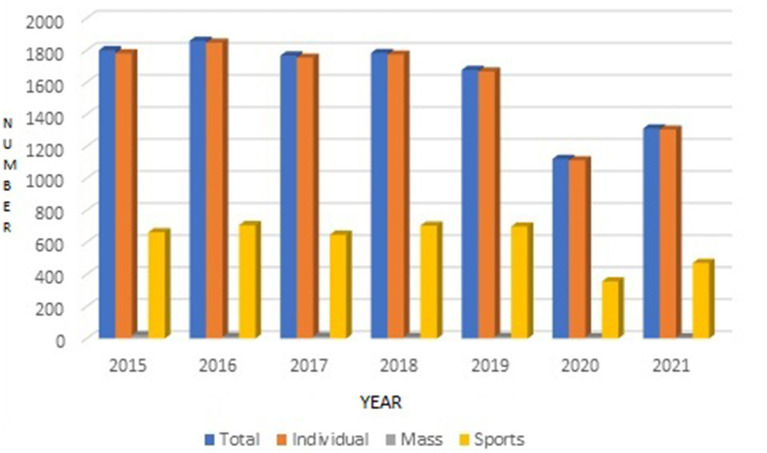
Total number of accidents of fire officers on duty, including sports injuries.

During the observation period, about 30,000 officers were on duty in the SFS, of which about 20% were on daily (8-h) duty, and 80% were on shift (24-h) duty (Figure 1).

Between 2015 and 2021, there were *N* = 11,332 (Mean: 1617.4; SD: 284.1) accidents in SFS (Figure 1). The accident total covers individual injuries and mass accidents. The number of sports injuries was *N* = 4,254 (Mean: 532.2; SD: 137.9) and accounted for 37.53% of the total. This group of incidents also covered individual and group accidents, e.g., soccer accidents – jumping to claim an overhead pass and the heads of two firefighters (a striker and a defender from opposing teams) clashing.

**Figure 2:**
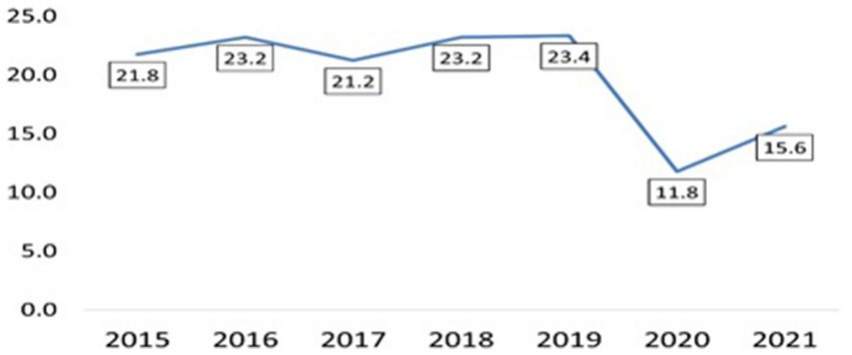
Total number of sports accidents among firefighters in Poland in 2015–2021 (data per 1,000 firefighters).

**Figure 3:**
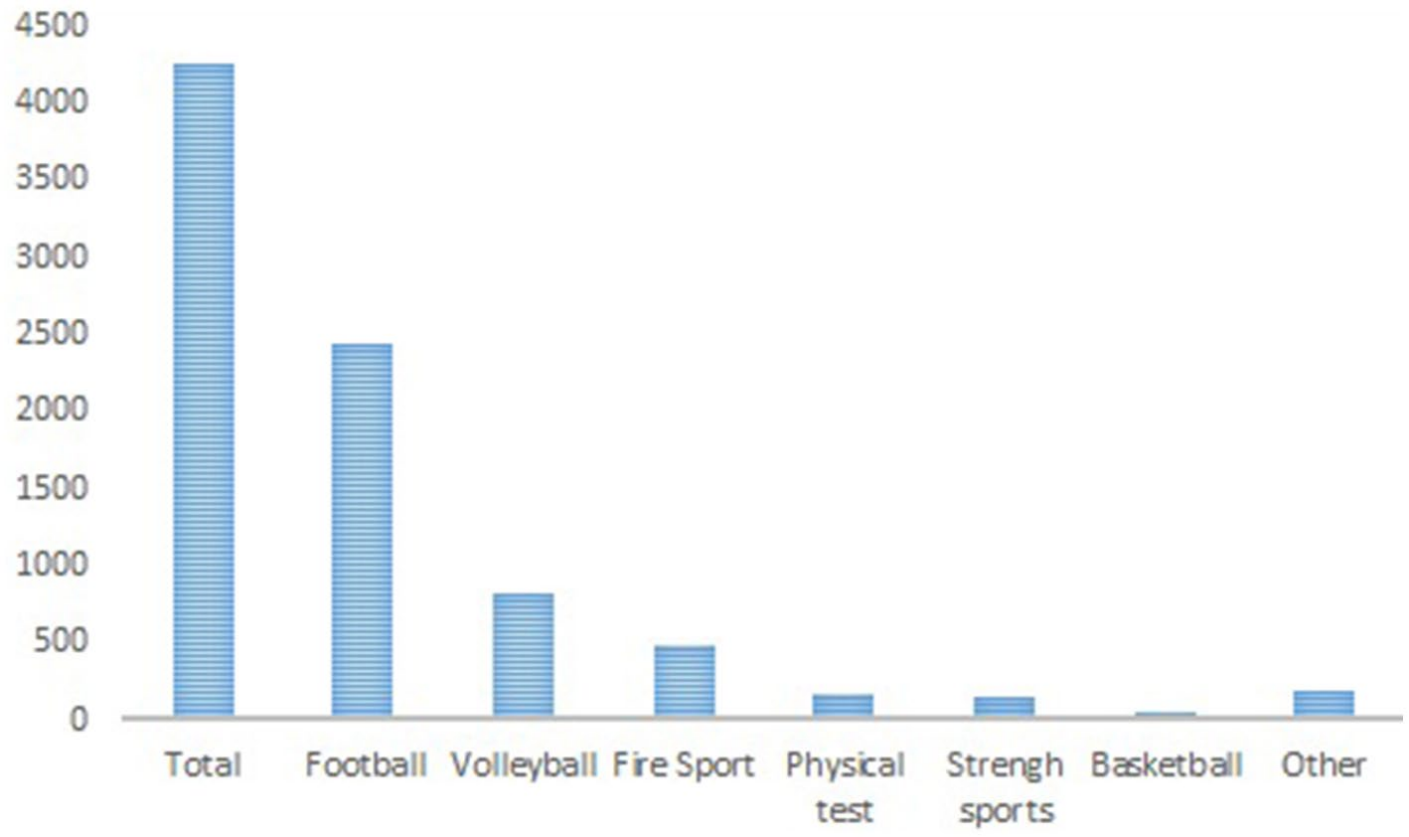
Participation of individual sports disciplines in injuries of firefighters in 2015–2021.

Figure 2 depicts a graphical representation of the number of sports accidents per 1,000 employed firefighters in subsequent years, without consideration for the type of sport. This allows for the identification of trends over time. In contrast, Table 2 provides a comparative analysis of the average number of sports accidents per 1,000 employed firefighters across selected sport disciplines. The data is statistically evaluated using an analysis of variance (ANOVA) (Figure 3).

**Table 2:** Average number of sports accidents in sports disciplines among firefighters in Poland in 2015–2021 (data per 1,000 firefighters).

Discipline	2015		2016		2017		2018		2019		2020		2021		*p*-value
	M	SD	M	SD	M	SD	M	SD	M	SD	M	SD	M	SD	
Football	12.3	5.40	13.6	5.94	11.9	5.19	13.6	5.66	13.3	4.32	6.7	4.11	9.0	3.63	<0.001
Volleyball	4.5	2.34	4.8	3.67	4.2	1.92	4.4	2.56	3.8	1.97	2.9	1.66	3.1	1.68	0.197
Physical test	1.5	1.21	1.4	0.92	1.2	0.91	1.1	0.91	0.8	0.78	0.0	0.11	0.0	0.12	<0.001
Fire sports	4.2	2.71	3.7	2.60	3.0	1.69	3.3	2.56	3.5	2.72	0.4	1.02	0.9	1.19	<0.001
Other*	1.3	0.85	1.5	1.09	2.4	2.11	1.9	1.16	2.2	1.35	1.8	1.34	2.4	2.30	0.319

*P*-value- one-way ANOVA. *Basketball, table tennis, strength sports, darts, billiards, floorball, jogging, swimming, tennis, badminton, treadmill.

The largest number of health risks is associated with team sports: football 57.16% and volleyball 19.06%, much less basketball (1.01%), which may be related to the lower popularity of this discipline in Poland (Figure 3).

The section “Other” includes individual cases of injuries related to sports activities that were not assigned to specific categories in the database.

**Figure 4:**
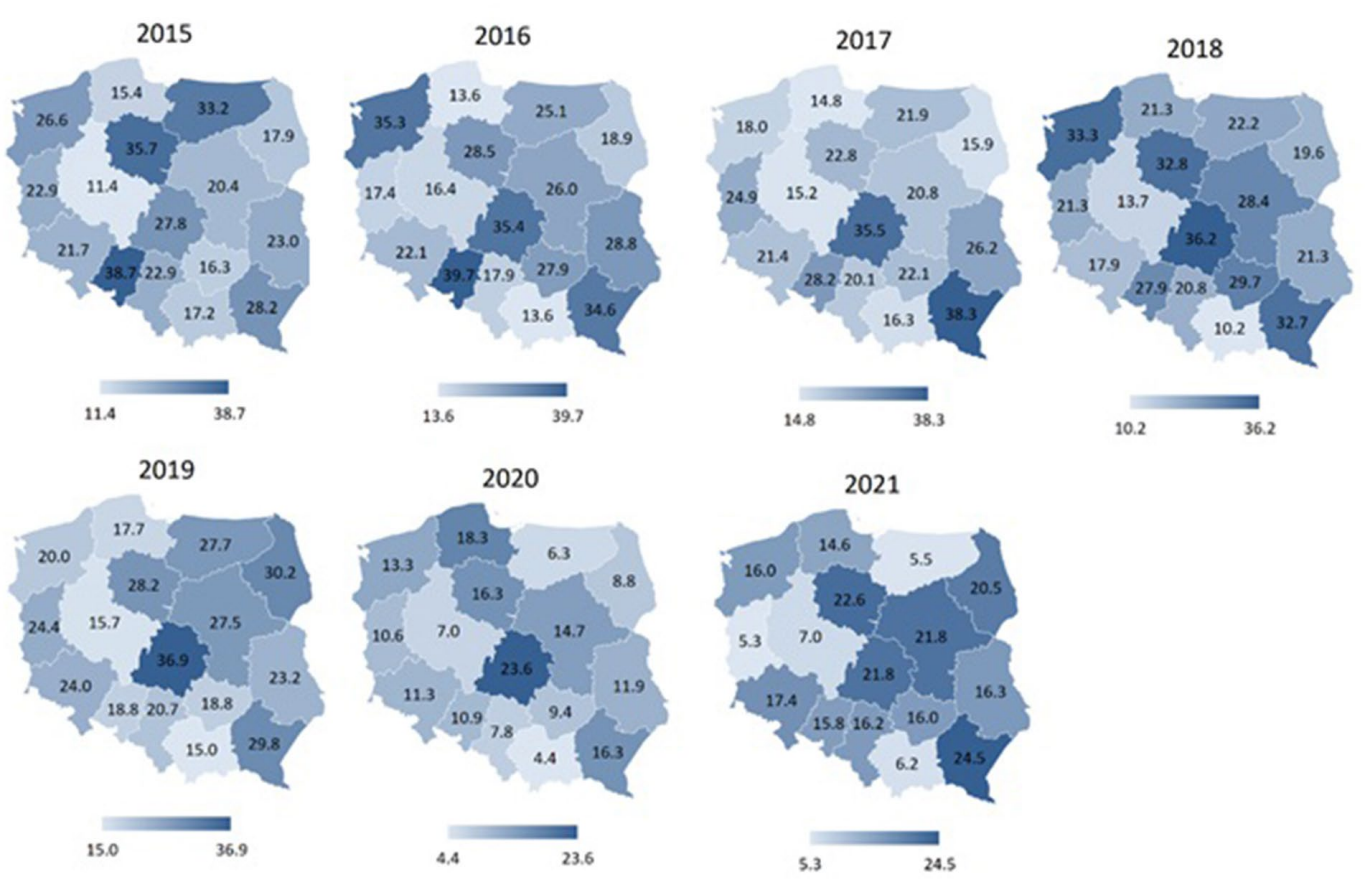
Total number of sports accidents among firefighters in different regions of Poland in 2015–2021 (data per 1,000).

**Figure 5:**
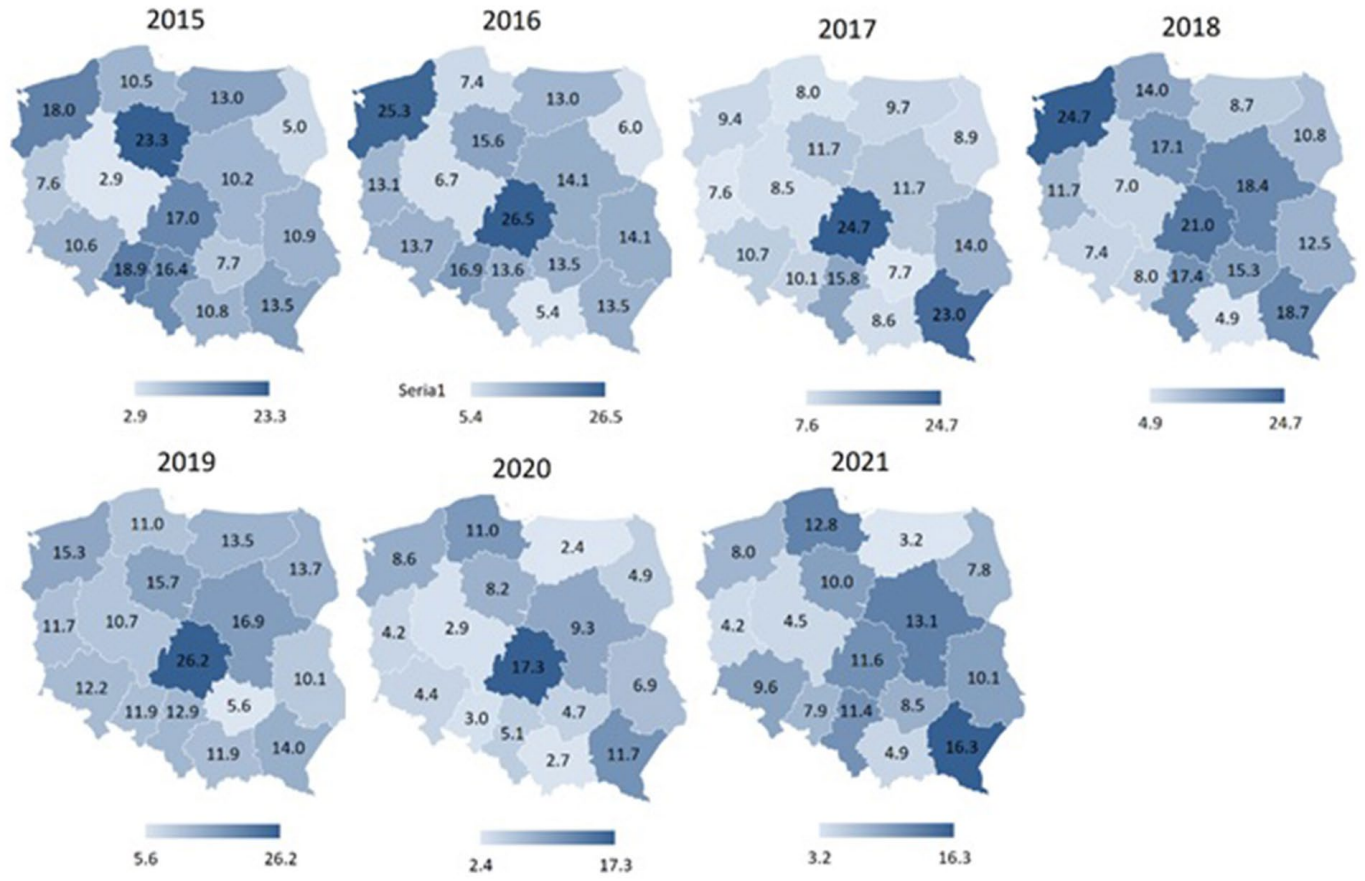
Number of football accidents among firefighters in different regions of Poland in 2015–2021 (data per 1,000).

**Figure 6:**
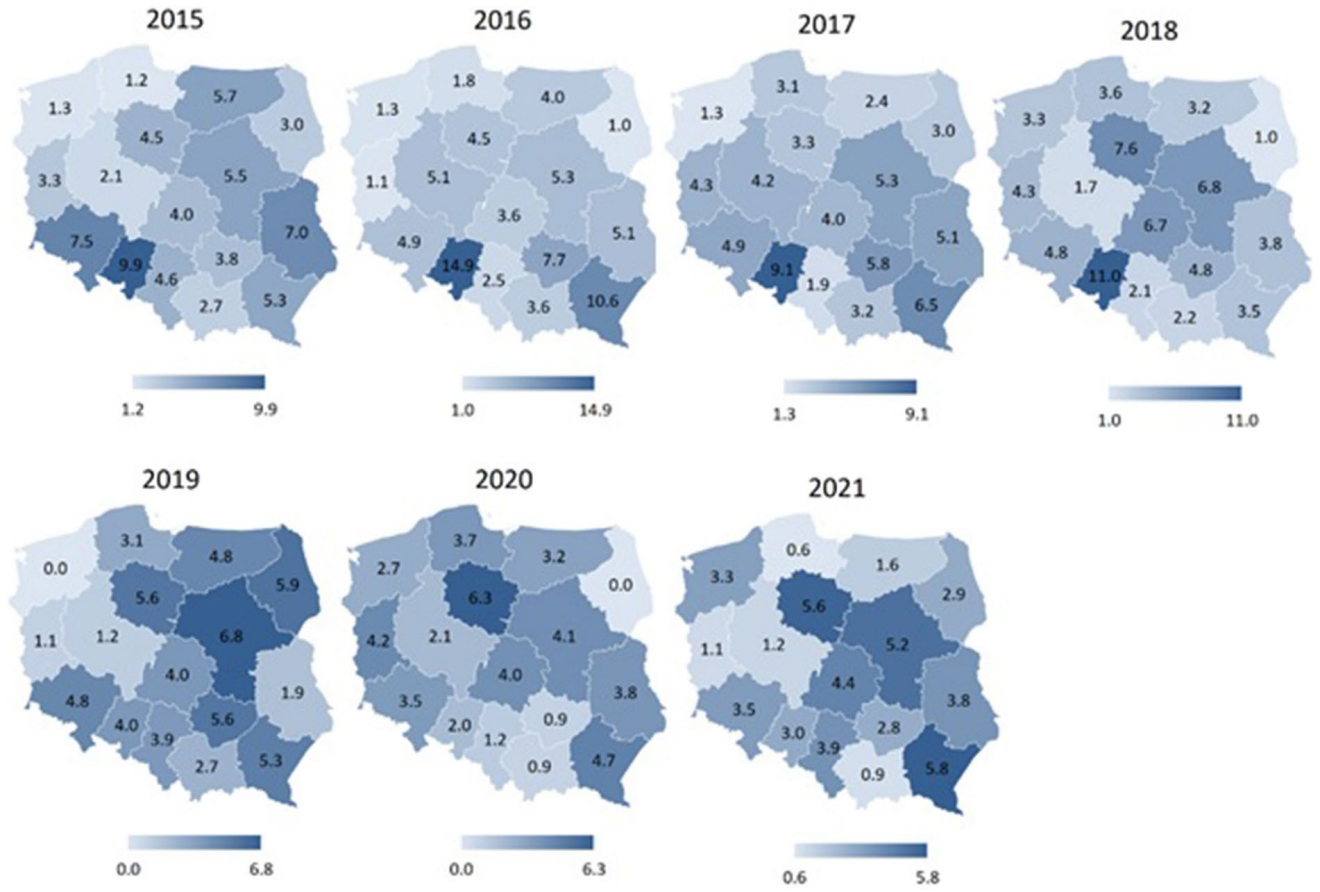
Number of volleyball accidents among firefighters in different regions of Poland in 2015–2021 (data per 1,000).

**Figure 7:**
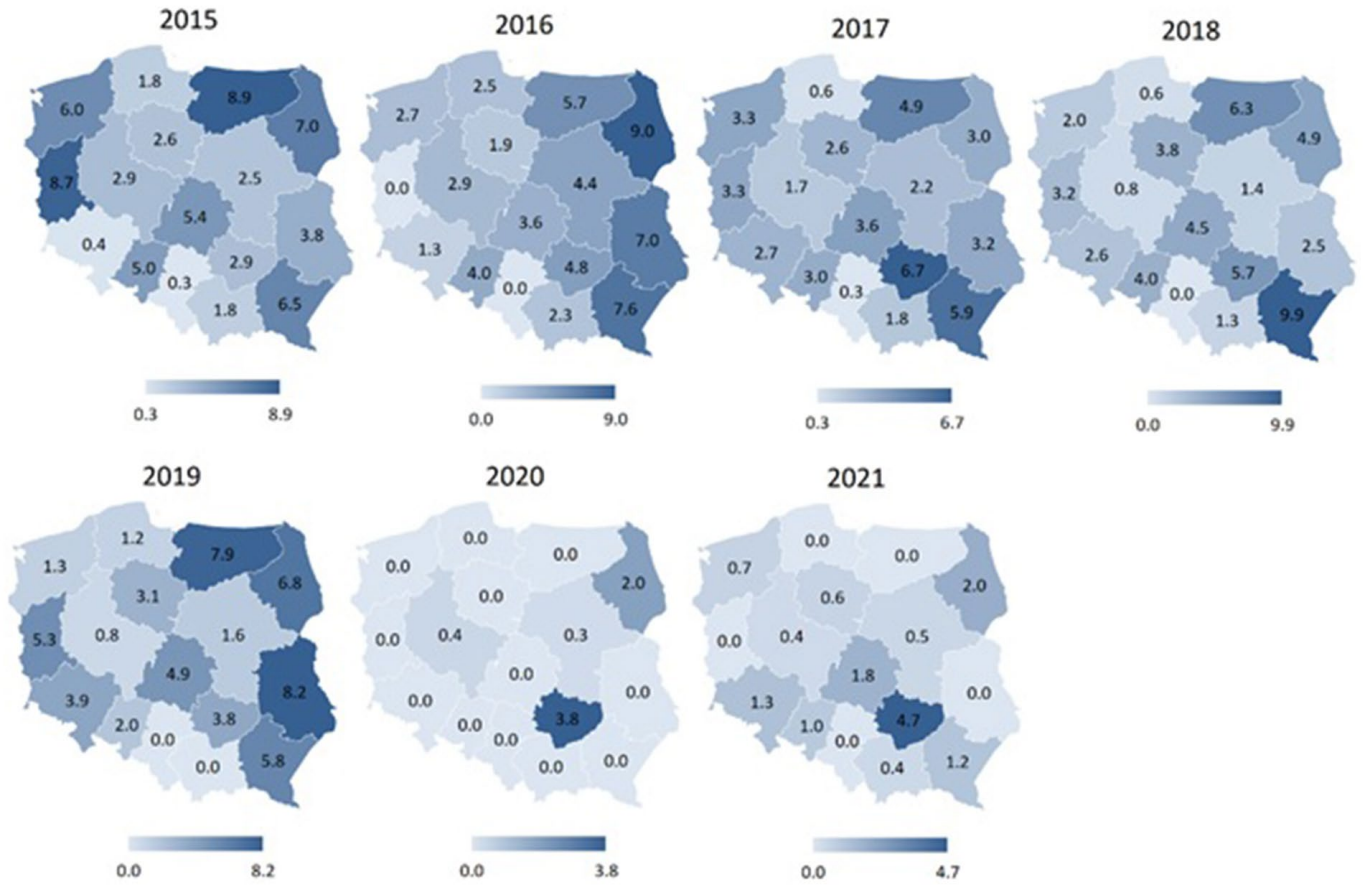
Number of fire sports accidents among firefighters in different regions of Poland in 2015–2021 (data per 1,000).

Figures 4–7 display spatial characteristics for the regions of Poland with respect to total accidents and 3 selected sports disciplines that generate the most firefighter accidents. The intensity of the phenomenon presented in the figures illustrates the number of sports accidents in each province in each year of observation. Particularly noteworthy is the year 2020 nationwide for fire sports. Accident rate in this sport discipline has fallen to 0 in many provinces, due to restrictions on sports competitions in the wake of the SARS-CoV-2 epidemic.

### Discussion

Sports injuries account for a significant share of all service-related firefighter accidents. This is due in part to the importance of sports in the profession of firefighting, the number of officers participating in various competitions and the ambition of the competitors wanting to achieve the best possible result in sports competitions. Team sports (soccer, volleyball, and fire sport, which is a multi-stage, technically difficult competition carried out under time pressure, highly competitive with athletes doing their best to achieve the best possible result), proved to be the most injury-prone.

Fire sport verifies the fitness of firefighters in the conditions, which are similar to conditions present in rescue and firefighting operations and it show the specifics of such operations. It requires a lot of physical effort, speed of action, reflexes, poise and teamwork skills. The injury rate for this type of firefighters’ sports activities is relatively high at 11.28%, second only to the popular team sports: soccer 57.16% and volleyball 19.06%. The performance of tasks (i.e., particular events) of fire sport in real-time competition (several teams perform simultaneously), time pressure, and fighting for the best possible result make injuries quite common. This discipline includes 4 competitions:

- fire obstacle race – a 100-meter run, including overcoming a wall, unrolling and connecting 2 sections of fire hose, overcoming a balance beam, connecting a hose line to fire fittings,
- climbing on the third floor (sham building) using a hook ladder,
- 4×100-meter relay with obstacles, including running on the roof of a sham house, overcoming a wall, balance beam, making a hose line and putting out a fake fire, (interestingly, and as a variation on the relay known from athletic competitions, the baton is a fire hose nozzle),
- main task- a team of 7 firefighters is tasked with forming a suction line, sucking water from a pool and filling a 10-liter tank through pouring holes in the discs (19).

An example of a situation that led to injury in the fire sport discipline: while performing the main task, during the stage of sucking water from the pool, the flexible fire hose W110 fell out of the firefighter’s hands, the hose “snapped” due to the high pressures generated by the fire pump and the metal connector of the hose hit the competitor in the face causing injury to the upper jaw (cut, bleeding, swelling). The cause of accident – carelessness, time pressure.

The group accident mentioned in the Results section: during a game of soccer, the officers clashed their heads while jumping for the ball. Both of the injured officers suffered cuts to the scalp in the frontal area. The cause of accident: team sport, contact sport, fighting for the ball.

An important issue in responding to injuries of firefighters is providing medical assistance. Firefighters are prepared to carry out medical activities at the level of Qualified First Aid (QFA), so in any case of injury to a firefighter, it is the colleagues who provide assistance first, while waiting for the arrival of the Emergency Medical Team (EMT), if the injured person’s condition requires it (20–22).

Polish and foreign literature list several works on the physical fitness of firefighters, and the influence of fitness on the effective implementation of rescue operations. Garano (23) showed that ball-based team sports played by firefighters while on duty bring financial consequences to the system in the form of prolonged indisposition of injured firefighters, the need for replacements, as well as injury compensation for injured officers. In some cases, an injury suffered during a ball game may be the reason for early retirement. Self-observation revealed, that ball-related sports were the most injury-prone.

There was also made an interesting observation about women in the fire service, where the authors refer to the issue of physical fitness. Higher overall self-assessment of health and fitness were associated with lower likelihood of sustaining an injury, while poor fitness level among firefighters can increase the risk of injury and jeopardize professional readiness (24). The author’s own study did not include a gender breakdown.

Jafari (25) found that lower level of physical fitness increases the risk of sustaining an injury, so improving functional fitness reduces the risk of injury in rescue operations. Similar conclusions were drawn by Cornell (26), noting a link between injuries and physical fitness. Nearly half of the injuries experienced by firefighters are musculoskeletal injuries, and the risk is determined by the functional quality of movement while on duty (e.g., during tasks requiring one-leg movement). Physical development and physical fitness gained during physical activity of firefighters reduces the risks during operations.

Morris (27) did not estimate the number of injuries, but showed that the physical fitness of firefighters is one of the requirements of the profession, and the reaction time of an athletic firefighter has an impact on firefighting operations and critical situations.

In domestic studies, several authors have highlighted sports-related injuries in the firefighter population. The 2020 study covering 2008–2013 showed that the greatest number of firefighter injuries was a result of sports activities. The predominant cause of incidents was improper demeanor or carelessness. The most common injuries from accidents were multiple fractures, broken bones and dislocations (28).

A 2020 survey questionnaire examined the most common health risks while on duty among firefighters. The most common sports injuries came from accidents were multiple fractures, broken bones and dislocations. However, being careful is not the only factor that determines safety during sports activities. Performing a warm-up that is appropriate for a given activity is of great importance, as well as the facilities and sports equipment that is used for the exercises (29).

Wejman points to various causes of occupational injuries sustained by firefighters; among them, overloading the musculoskeletal system as a result of physical activity (30). Wicherek-Brzozowska shares the view of other researchers, namely, that the right level of physical fitness is in many cases a prerequisite for a certain professional role. Firefighters act in situations that require sharp thinking and prudence, quick reaction and high mental and physical endurance (31).

Kozłowski et al. compared the injury rate of professional firefighters and volunteers related to physical activity. An ankle sprain was a common injury in both groups. Our study did not look at volunteer fire departments. However, the injury mentioned by the researchers is common in contact sports (football and volleyball), which may be confirmed in our analysis as well as in other studies (32–34).

According to the authors of the study, a noticeable decrease in sports injuries occurred in 2020 (175 less than the average) and 2021 (60 less than the average). They speculate that this may be related to the restrictions caused by the SARS-CoV-2 pandemic. For epidemiological reasons, the annual calendar of sports competitions in the SFS has been significantly limited, and a large number of competitions (mainly team ones) have been suspended at every administrative level (district, province). However, the conclusion is derived exclusively from circumstantial evidence, thus precluding the definitive establishment of a causal relationship between the events in question (35).

## Limitations

Our analysis covers the entire population of Polish professional firefighters, including a large number of injuries. But it has some limitations. The analysis does not account for sports injuries sustained by volunteer firefighters and by fire protection units’ civilian staff for whom there are also sports competitions organized in many events at various levels. This group also suffers sports-related injuries while representing firefighting units. In addition, the authors did not have access to the clinical (hospital) documentation of firefighters’ injuries (medical procedures in the hospital, imaging diagnostics performed, diagnosis code according to the ICD-10 standard, length of treatment, time off duty as a result of the injury). Socio-demographic data: age of firefighters, gender, type of injuries were available in the reports, but in the form of separate calculations not correlated with each other. Hence, we know the average age of a firefighter who suffered sports accidents, but without the possibility of assigning age, seniority, job position to a specific injury in the described sports discipline.

## Conclusion

There is a need for comprehensive approach to physical training in the firefighter population. Physical activity should be continuous and systematic strengthening of the whole body. There should be constant and consistent monitoring of physical activity performed by officers exerted by managers of SFS organizational units. Consideration should be given to conducting in-service training for supervisors, coaches and instructors in the safe conduct of sports activities. Sports activities should begin with performing thorough warm-ups. It is necessary to maintain facilities, premises, equipment and technical devices in a condition that sets the ground for doing sports safely and in a hygienic manner. Most of the sports injuries sustained by firefighters are related to team sports.

## Data availability statement

The raw data supporting the conclusions of this article will be made available by the authors, without undue reservation.

## Ethics statement

Ethical review and approval was not required for the study on human participants in accordance with the local legislation and institutional requirements. Written informed consent for participation was not required for this study in accordance with the national legislation and the institutional requirements.

## Author contributions

ŁD: conceptualization, data curation, methodology, investigation, manuscript preparation, and approved the final version. MP: formal analysis, investigation, and approved the final version. TK: visualization and approved the final version. TM: data curation, investigation, and approved the final version. All authors contributed to the article and approved the submitted version.

## Conflict of interest

The authors declare that the research was conducted in the absence of any commercial or financial relationships that could be construed as a potential conflict of interest.

## Publisher’s note

All claims expressed in this article are solely those of the authors and do not necessarily represent those of their affiliated organizations, or those of the publisher, the editors and the reviewers. Any product that may be evaluated in this article, or claim that may be made by its manufacturer, is not guaranteed or endorsed by the publisher.
